# Orbital Complications of Acute Rhinosinusitis in Adulthood: Predictors of Outcome and Management

**DOI:** 10.1002/lary.70309

**Published:** 2025-12-12

**Authors:** Alessandro Vinciguerra, Vittorio Rampinelli, Mario Turri‐Zanoni, Marco Ferrari, Marco Valentini, Alberto Daniele Arosio, Federico Raimondi, Florian Chatelet, Stefano Taboni, Davide Mattavelli, Alberto Schreiber, Fabio Sovardi, Matteo Barucco, Lorena Di Girolami, Piergiorgio Gaudioso, Antonio Daloiso, Giulia Danè, Umberto Tanzini, Nicola Tessari, Jessica Zuppardo, Kays Burak Cakir, Yetkin Zeki Yilmaz, Yasar Unlu, Andrea Ronchi, Stefania Gallo, Francesca De Bernardi, Vasileios Chatzinakis, Argyro Leventi, Fabio Pagella, Benjamin Verillaud, Giuseppe Mercante, Apostolos Karligkiotis, Mario Bussi, Cesare Piazza, Alperen Vural, Paolo Castelnuovo, Piero Nicolai, Maurizio Bignami, Christos Georgalas, Iacopo Dallan, Philippe Herman, Paolo Battaglia

**Affiliations:** ^1^ Division of Otorhinolaryngology, Department of Biotechnology and Life Sciences University of Insubria, ASST Lariana Como Italy; ^2^ Unit of Otorhinolaryngology—Head and Neck Surgery, ASST Spedali Civili Brescia, Department of Medical and Surgical Specialties, Radiological Sciences, and Public Health University of Brescia Brescia Italy; ^3^ Unit of Otorhinolaryngology—Head and Neck Surgery Azienda Ospedale‐Università Padova Padova Italy; ^4^ Section of Otorhinolaryngology—Head and Neck Surgery, Department of Neuroscience (DNS) University of Padova Padova Italy; ^5^ Division of Otorhinolaryngology, Department of Biotechnology and Life Sciences University of Insubria, Ospedale di Circolo Varese Italy; ^6^ Respiratory Medicine Unit ASST Papa Giovanni XXIII Bergamo Italy; ^7^ Otorhinolaryngology and Skull Base Center, AP‐HP, Hospital Lariboisière Université Paris Cité Paris France; ^8^ Department of Otorhinolaryngology Fondazione I.R.C.C.S. Policlinico San Matteo Pavia Italy; ^9^ Skull Base and Rhino‐Orbital Surgery Unit Azienda Ospedaliero‐Universitaria Pisana Pisa Italy; ^10^ Otorhinolaryngology Unit, Division of Head and Neck Department IRCCS San Raffaele Scientific Institute Milano Italy; ^11^ Department of Otorhinolaryngology—Head and Neck Surgery IRCCS Humanitas Research Hospital Milan Italy; ^12^ Department of Biomedical Sciences Humanitas University Milan Italy; ^13^ Department of Otorhinolaryngology, Cerrahpasa Faculty of Medicine Istanbul University—Cerrahpasa Istanbul Turkey; ^14^ Department of Otorhinolaryngology, Faculty of Medicine Erciyes University Kayseri Turkey; ^15^ Medical School University of Nicosia Nicosia Cyprus

**Keywords:** acute sinusitis, Chandler classification, orbital cellulitis, orbital infection, pre‐septal cellulitis

## Abstract

**Objectives:**

Orbital infections of acute rhinosinusitis are commonly classified thanks to the Chandler classification and may lead to vision loss or diplopia if not properly managed. While pediatric cases are well documented and their management is supported by clear evidence, data guiding adult management remain limited and fragmented. This study analyzes a large international cohort of patients that evaluates treatment approaches and outcome predictors for orbital complications (OCs) of acute sinusitis in adulthood.

**Methods:**

This multicentric retrospective study included adults with OCs of acute sinonasal infections. Patients were classified using the Chandler classification, with an additional subdivision for pre‐septal infections (modified Chandler classification). Clinical, radiologic, and therapeutic data were analyzed, evaluating treatment success, hospital stay, and complications. Predictors of treatment and outcomes were studied (*p* < 0.05).

**Results:**

Among 213 patients (65.3% male, median age 48), 68.2% required surgery, mainly endoscopic (60.7%). Logistic regression identified the presence of additional complications (*p* = 0.015) and modified Chandler classification (*p* < 0.001) as the strongest predictors for treatment modality, while sinus opacification and visual impairment lost significance in the multivariate model. Infection resolution after primary treatment was significantly associated with nasal corticosteroid use (*p* = 0.037). Despite differences in treatment approach and hospitalization duration across modified Chandler categories, no significant differences were observed in final ophthalmologic outcomes.

**Conclusion:**

This study emphasizes the role of the modified Chandler classification for upfront treatment decisions. Abscess‐related and type II OCs often needed surgery, yet all cases achieved similarly optimal ophthalmologic outcomes and final infectious resolution.

**Level of Evidence:**

4.

## Graphical abstract

In a multicenter cohort of 213 adults with orbital complications of acute rhinosinusitis, 68% required surgery and the modified Chandler classification was the strongest predictor of treatment modality. While abscess‐related and type II complications frequently necessitated surgical management, final ophthalmologic outcomes were uniformly favorable across groups. Nasal corticosteroid use was independently associated with improved infection resolution after primary treatment.

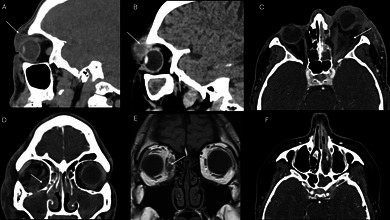

## 1 Introduction

Orbital involvement is the most common complication of sinonasal infections and can be the result of isolated acute rhinosinusitis (ARS) or acute exacerbation of chronic rhinosinusitis. Usually, this infection spreads to the orbital cavity directly through neurovascular foramina, congenital/acquired dehiscence, or through a hematogenous spread, known as infectious thrombophlebitis [1, 2]. Among all paranasal sinuses, the ethmoidal sinus appears to be the most involved, while the frontal sinus is more often associated with intracranial complication and osteitis [3]. Predisposing factors for orbital complications (OCs) are represented by immunocompromised status (either acquired or congenital), impaired sinus drainage and inhibition of mucociliary transport, which may promote bacterial overgrowth [4, 5].

In 1970, Chandler et al. [6] proposed a progressive classification of orbital involvement of sinonasal infection based on radiological and clinical findings: type I, pre‐septal oedema (cellulitis or abscess); type II, orbital cellulitis; type III, subperiosteal abscess; type IV, orbital abscess; type V, cavernous sinus thrombosis, a rare condition (< 1%) with high mortality rate (20%–30%) [7]. In 1997 and 2007, two different groups proposed new classifications criticizing the previous inclusion of cavernous sinus thrombosis and pre‐septal infection in the group of OCs, since both anatomically sit outside the orbital cavity [3, 8]. However, even if those authors presented respectable arguments with meaningful anatomical principles, the Chandler classification is still the most commonly applied worldwide, owing to its simplicity and longstanding [9].

Appropriate management of these OCs is of paramount importance since, if not properly addressed, they may result in permanent vision impairment, brain abscess, meningitis, and possibly death [10]. However, differently from the pediatric population, in which the literature presents solid references about the correct approach for each of the above‐mentioned OC types [11, 12, 13], adult management is based on sporadic monocentric case series or administrative database analysis. Specifically, it is believed that the pre‐septal involvement and orbital cellulitis respond to exclusive medical treatment, with surgery reserved for subperiosteal/orbital abscess [1]. Nevertheless, these therapeutic choices are not supported by robust scientific evidence leaving the appropriateness of the medical and/or surgical approach for each type of orbital infection in adulthood open to debate [10].

The aim of this manuscript is to present a large, international, multi‐institutional, real‐world cohort of adult patients with orbital involvement from ARS, with a primary focus on identifying predictors of management strategies and clinical outcomes across the different types of OCs.

## 2 Methods

This is a multicentric retrospective observational study performed at nine tertiary care referral centers.

Inclusion criteria were: (a) Adult patients (age > 18 years); (b) patients with OCs secondary to acute sinonasal infection; (c) patients managed either conservatively (exclusive medical therapy) and/or surgically; (d) minimum follow‐up of 3 months; and (e) patient treated between January 2010 and December 2024. Exclusion criteria were patients who experienced an orbital involvement because of invasive mycotic infections.

Informed consent was obtained from each patient for treatment and use of deidentified clinical data for study purposes; the study was conducted according to the ethical standards of the Declaration of Helsinki revised in 2011 and approved by all centers review boards.

### 2.1 Patient Management and Study Variables

In all cases, the diagnostic work‐up included a preoperative radiological study (computed tomography [CT] and/or contrast‐enhanced magnetic resonance imaging [MRI]).

**FIGURE 1:**
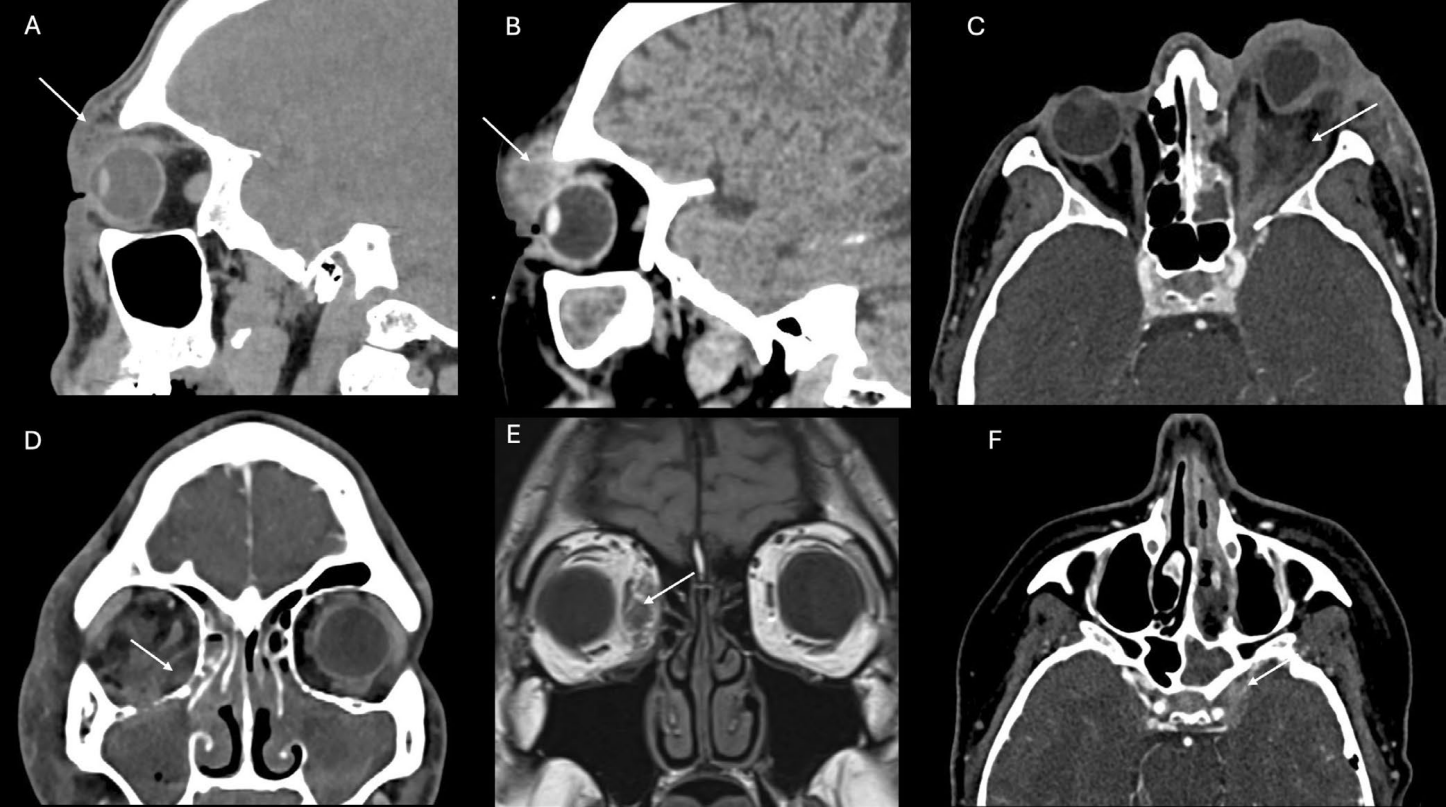
Radiologic representations of patients affected with different type of Chandler infections. (A) Left pre‐septal cellulitis (white arrow) of a patient with acute frontal sinusitis (type Ia); (B) left pre‐septal abscess (white arrow) of a patient with left odontogenic fronto‐ethmoidal‐maxillary acute sinusitis (type Ib); (C) left orbital cellulitis (white arrow), with significant proptosis, for an acute ethmoidal sinusitis (type II); (D) right subperiosteal abscess (white arrow) for an acute infection of chronic rhinosinusitis with nasal polyps (type III); (E) right intraconal abscess (white arrow) for an ethmoidal acute sinusitis treated conservatively (type IV); and (F) left cavernous sinus thrombosis (white arrow) for a left fronto‐ethmoidal‐sphenoidal sinusitis (type V).

Given that classifications aim to facilitate communication among clinicians and that the original Chandler classification [6] remains the most widely used [14], our cases were categorized accordingly. However, based on the observation of different treatment approaches between cellulitis and abscess in pre‐septal infections (Chandler I), this category was a priori subdivided into Chandler Ia (cellulitis) and Chandler Ib (abscess) and analyzed accordingly (Table 1, Figure 1).

**TABLE 1:** Comparison between the original Chandler classification and its modified version.

Description	Chandler's classification	Modified Chandler's classification
Pre‐septal cellulitis	I	Ia
Pre‐septal abscess		Ib
Orbital cellulitis	II	II
Subperiosteal abscess	III	III
Intraconal orbital abscess	IV	IV
Cavernous sinus thrombosis	V	V

Data considering the treatment (both medical and surgical) of all OCs were collected and analyzed considering the success of treatment, duration of antibiotics (ATBs), days of hospitalization, aggressiveness of treatment (i.e., application of a drain inside the involved pathological sinus and/or orbital abscess) and outcomes. All surgically treated patients were instructed to perform isotonic saline nasal irrigations twice daily starting from the first postoperative day and continuing for at least two weeks. Follow‐up visits were based on in‐office endoscopic evaluation every 2–3 weeks and personalized on each patient [15]; a postoperative radiological exam was performed if considered necessary (i.e., suspicion of iatrogenic mucocele formation).

Data on age, sex, laterality of the infection, symptoms referred, comorbidities, additional complications (referred to other forms of complicated ARS associated with the orbital infection, including intracranial extension such as abscess/meningitis/cerebritis, osteomyelitis, and septic ischemic stroke) leukocytosis, period of the year of the infection, radiological aspect of involved sinus, and follow‐up updated in December 2024 were also collected.

Primary endpoints of this study were to identify predictors of management strategies and clinical outcomes in adult patients with OCs of ARS, while also evaluating the role of the modified Chandler classification.

### 2.2 Statistical Analysis

Statistical analyses were conducted using SPSS (version 24, IBM Corp., Armonk, NY, USA). A normality test was applied for continuous variables which were treated accordingly; the Kruskal–Wallis test was used in non‐normally distributed variables to compare multiple independent groups, with Bonferroni‐adjusted pairwise comparisons when significant. Categorical variables were analyzed using the *χ* ^2^ or Fisher's exact test; Cramer's *V* value was calculated to assess the strength of association between categorical variables. To identify predictors of treatment choice and clinical outcomes, logistic regression models were applied in both univariate and multivariate analyses. *p* < 0.05 was considered statistically significant.

## 3 Results

In total 213 patients were included in this study, with 64.4% males and a median age of 48 years (IQR 28–63). Most cases affected the left side (55.4%) and leukocytosis was observed in 52.2% of cases, with a median leukocyte count of 13.79 10^3^/mL (IQR 12–15.73) (Table 1). The median time from nasal symptom onset to hospital referral was 5 days, while for ophthalmologic symptoms, it was 3 days. Radiologic sinus involvement was often combined rather than isolated. The most frequent patterns were ethmoido–fronto–maxillary (33.6%) and pansinusitis (22%), followed by ethmoido–maxillary (10.3%) and ethmoido–frontal (9.3%). Isolated involvement was less common, observed in 3.3%–5.6%.

A comparative analysis between the original and the modified Chandler classification was performed to assess the predictive value of the revised classification. The likelihood ratio test showed a significant improvement in model fit for the modified classification compared with the classic one (ΔDeviance = 5.0; df = 1; *p* = 0.025), consistent with lower AIC (147 vs. 150) and BIC (176 vs. 177), higher Nagelkerke's *R* ^2^ (0.463 vs. 0.439), and a slightly higher AUC (0.850 vs. 0.841). These results support the validity and clinical usefulness of the modified classification in the adult population.

When considering treatment modalities, 68.2% of patients required a surgical approach, while 31.8% were managed conservatively (Table 2). Among surgical cases, endoscopic surgery was the preferred approach (60.7%), while exclusive external surgery was performed in 4.8% of cases; a combined surgery was applied in 34.5% of cases. Specifically, in all endoscopic surgically treated cases, a complete ethmoidectomy and middle antrostomy (eventually extended to the frontal and/or sphenoidal sinus based on preoperative radiologic imaging) were performed; if necessary, the OC was drained surgically either through an endoscopic or external approach, based on its location. Drainage inside the pathologic sinus was placed in 23.4% of cases, whereas inside the orbital abscess in 30.1%.

**TABLE 2:** General clinical characteristics of patients involved in the study.

Variable	Value, *N* = 216
Age (years), median (IQ)	48 (28–63)
Sex, *N* (%)	
Female	74 (34.7)
Male	139 (65.3)
Side, *N* (%)	
Right	95 (44.6)
Left	118 (55.4)
Immunocompromised, *N* (%)	
No	186 (87.3)
Yes	27 (12.7)
Comorbidities (other than immunodeficiency), *N* (%)	
No	148 (69.4)
Yes	65 (30.6)
Leucocytosis, *N* (%)	
No	96 (47.8)
Yes	105 (52.2)
No. leucocytosis, median (IQ)	13.79 10^3^/mL (12–15.73)
Radiologic exams performed, *N* (%)	
CT	144 (70.2)
MRI	11 (5.4)
CT + MRI	50 (24.4)
Radiologic sinus opacification, *N* (%)	
Partial	52 (26)
Complete	131 (65.5)
Air bubbles	17 (8.5)
Abscess Width (mm), median (IQ)	16.6 (9–27)
Underlying CRS, *N* (%)	
No	121 (56.8)
Yes	92 (43.2)
Odontogenic infection, *N* (%)	
No	173 (81.2)
Yes	40 (18.8)
Days between nasal symptom onset and hospital presentation, median (IQ)	5 (2–5)
Days between ophthalmologic symptom onset and hospital presentation, median (IQ)	3 (1–3)

Exploratory descriptive analysis showed that several clinical variables significantly differed across modified Chandler classification subgroups (Table 3). A strong association was found between the modified Chandler classification and treatment strategies (*p* < 0.001, Cramer's *V* = 0.518). Specifically, with type Ia, most patients (69%) were managed medically; type Ib had a more variable approach, with a notable increase in surgical management (87.5%) compared to Ia; type II patients demonstrated a mixed profile, 30.8% treated medically and 69.2% surgically, with 9 cases of medial orbital decompression, and 16 endoscopic drainages of the endonasal infection; type III–IV showed a clear shift toward surgical approach compared to type II, with, respectively, 86.8% and 88.2% of patients requiring at least one surgical procedure (with either endoscopic or external drainage of the abscess based on its location). The number of surgeries varied significantly among classes (*p* < 0.001, Cramer's *V* 0.567), with the type Ib being the most commonly operated ≥ 2 times (37.5%), either for an endoscopic or external drainage. The hospitalization and ATBs durations varied among all OC types (*p* < 0.001).

**TABLE 3:** Association between Chandler's infections and their management with *χ* ^2^ or Kruskal–Wallis test. *p* Values are shown in linear‐by‐linear association.

	Modified Chandler classification						
	1a	1b	2	3	4	5	*p* (Cramer's *V*)
No. of cases, total of 213 (%)	58 (27.2)	8 (3.8)	39 (18.3)	55 (25.9)	51 (23.9)	2 (0.9)	
Ophthalmologic pretreatment signs/symptoms, *N* (%)							0.501
None	22 (56.4)	2 (40)	5 (20.8)	7 (17.1)	14 (33.3)	0 (0)	
Ocular movement impairment	11 (28.2)	3 (60)	7 (29.2)	5 (12.2)	5 (11.9)	1 (50)	
Proptosis	0 (0)	0 (0)	7 (29.2)	14 (34.1)	15 (35.7)	0 (0)	
IOP > 23	0 (0)	0 (0)	0 (0)	0 (0)	1 (2.4)	0 (0)	
Visual acuity impairment	6 (15.4)	0 (0)	5 (20.8)	15 (36.6)	7 (16.7)	1 (50)	
Treatment, *N* (%)							**< 0.001 (0.518)**
Medical	40 (69)	1 (12.5)	12 (30.8)	7 (13.2)	6 (11.8)	1 (50)	
Surgical	18 (31)	7 (87.5)	27 (69.2)	48 (86.8)	45 (88.2)	1 (50)	
Number of total surgeries, *N* (%)							**< 0.001 (0.567)**
0	40 (68.9)	1 (12.5)	9 (23.1)	8 (14.5)	6 (11.8)	0	
1	16 (22.4)	4 (50)	24 (61.5)	42 (76.4)	37 (72.5)	2 (100)	
≥ 2	2 (7.3)	3 (37.5)	6 (15.4)	5 (9.1)	8 (15.7)	0	
ATBs therapy (days), median (IQ)							**< 0.01**
	13 (11–15)	14 (12–21)	15 (11–23)	14 (11–18)	16 (14–21)	35	
Hospitalization (days), median (IQ)							**< 0.001**
	5 (3–7)	8 (5–14)	6 (4–12)	6 (4–8)	8 (6–14)	15	
Resolution of the infection after primary treatment, *N* (%)							**0.03 (0.226)**
Yes	53 (91.2)	4 (57.1)	30 (78.9)	46 (90.2)	42 (85.7)	1 (50)	
No	5 (8.8)	3 (42.9)	8 (21.1)	5 (9.8)	7 (14.3)	1 (50)	
Final ophthalmologic outcomes, *N* (%)							0.745
Complete resolution	55 (94.7)	8 (100)	31 (86.1)	50 (94.3)	46 (92)	1 (50)	
Partial/no resolution	3 (5.3)	0 (0)	5 (13.9)	3 (5.7)	4 (8)	1 (50)	

*Note*: Significant results are reported in bold characters.

Abbreviations: ATB = antibiotics, IOP = intraocular pressure.

The most common primary ATB therapy was ceftriaxone + metronidazole (60 cases), amoxicillin/clavulanate (53 cases), ampicillin/sulbactam (35 cases), and levofloxacin (8 cases); the remaining 60 patients received various less frequent or personalized ATBs combinations (i.e., vancomycin, piperacillin/tazobactam, clindamycin).

After a median follow‐up of 10 months (IQR 4–24), no differences were noted among the modified Chandler categories considering the final ophthalmologic outcome, specifically for diplopia (four cases) and blindness (five cases), or intraoperative complications (*p* > 0.05).

### 3.1 Factors Influencing Primary Treatment (Table 4)

**TABLE 4:** Factors that influenced the primary treatment (medical vs. surgical) at univariate and multivariate analysis.

Variable	Univariate OR (95% CI)	*p*	Multivariate OR (95% CI)	*p*
Age	0.997 (0.983–1.111)	0.64	—	
Laterality	1.193 (0.670–2.126)	0.549	—	
Sex	0.718 (0.389–1.326)	0.290	—	
Immunocompromised disease	0.764 (0.330–1.770)	0.531	—	
Radiologic sinus opacification	2.467 (1.402–4.342)	**0.002**	1.814 (0.909–3.618)	0.091
Visual Impairment	3.000 (1.152–7.812)	**0.024**	2.387 (0.768–7.424)	0.133
Diplopia	3.097 (0.879–10.906)	0.066	—	
Presence of additional complications	8.105 (1.871–35.119)	**0.005**	5.891 (1.677–107.507)	**0.015**
Modified Chandler classification	2.166 (1.618–2.899)	**< 0.001**	1.743 (1.349–2.680)	**< 0.001**

*Note*: Significant results are reported in bold characters.

In the univariate analysis, the modified Chandler classification (OR 2.166, 95% CI 1.618–2.899, *p* < 0.001), radiologic sinus opacification (OR 2.467, 95% CI 1.402–4.342, *p* = 0.002), visual impairment (OR 3.000, 95% CI 1.152–7.812, *p* = 0.024), and presence of additional complications (OR 8.105, 95% CI 1.871–35.119, *p* = 0.005) were associated with a higher likelihood of surgical approach. When a multivariate model was applied, the strongest predictors for surgery were the presence of additional complications (OR 5.891, 95% CI 1.677–107.507, *p* = 0.015) and the modified Chandler classification (1.743 95% CI 1.349–2.680, *p* < 0.001); sinus opacification and visual impairment lost significance after adjustment.

### 3.2 Predictors of Infection Resolution After Primary Treatment (Table 5)

**TABLE 5:** Predictors of outcome (resolution of the infection) after primary treatment at univariate and multivariate analysis.

Variable	Univariate OR (95% CI)	*p*	Multivariate OR (95% CI)	*p*
Leucocytosis	0.768 (0.334–1.768)	0.535	—	
Chronic Rhinosinusitis (CRS)	1.066 (0.481–2.365)	0.875	—	
Immunocompromised disease	0.791 (0.250–1.264)	0.691	—	
Odontogenic infection	0.562 (0.219–1.444)	0.231	—	
Sepsis	0.158 (0.010–2.602)	0.197	—	
Preadmission NSAIDs	0.224 (0.099–0.508)	**< 0.001**	0.410 (0.153–1.099)	0.077
Nasal corticosteroids	4.211 (1.611–11.004)	**0.003**	3.028 (1.070–8.573)	**0.037**
Nasal vasoconstrictors	0.557 (0.171–1.818)	0.332	—	
Modified Chandler classification	0.838 (0.710–0.989)	**0.037**	0.870 (0.715–1.059)	0.163
Pus culture	0.388 (0.150–1.000)	**0.050**	0.327 (0.103–1.039)	0.059

*Note*: Significant results are reported in bold characters.

In the univariate analysis, intranasal corticosteroid (INCS) therapy (OR 4.211, 95% CI 1.611–11.004, *p* = 0.003), modified Chandler classification (OR 0.838, 95% CI 0.710–0.989, *p* = 0.037), and NSAID preadmission therapy (OR 0.224, 95% CI 0.099–0.508, *p* < 0.001) were significantly associated with infection resolution, however, when a multivariate model was applied, only INCS remained a significant predictor of resolution (OR 3.028, 95% CI 1.070–8.573, *p* = 0.037).

## 4 Discussion

The main findings of this study are: (1) the most important factor in guiding therapeutic decisions for ARS with OCs was the modified Chandler classification, with surgery reserved in most cases of type Ib–II–III–IV OCs, respectively, in 87.5%, 69.2%, 86.8%, and 88.2% (1.743 95% CI 1.349–2.680, *p* < 0.001); (2) INCS application represents the main protective factor in the resolution of the OC after primary treatment; (3) the therapeutic protocol applied in this large cohort of adult patients proved to be effective, so that the final ophthalmologic outcomes were comparable regardless of the type of initial orbital infection. To the best of the authors' knowledge, this paper represents one of the few articles in the literature that analyzes the management of orbital involvement in ARS in adulthood, with the largest case series ever described.

Orbital infections are commonly the secondary involvement of an untreated ARS and can present different ophthalmologic symptoms (i.e., diplopia, ophthalmoplegia, blindness) which are frequently the reason for hospital referral. Some authors suggested that the ophthalmological clinical presentation correlates with the OC type, for example, the incidence of elevated intraocular pressure is related to subperiosteal or intra‐orbital abscess and extra‐ocular movements are mainly affected in orbital cellulitis [1, 16]. However, in our series, the pretreatment ophthalmologic symptoms were not related to the OC, suggesting that the clinical presentation cannot be considered as a predictor for the type of orbital involvement and that radiologic exams (CT and/or MRI, performed in 94.5% of our cases) are of paramount importance to examine and classify the suspected orbital infection. Thanks to the radiologic exams performed, our series has been stratified through the most widely used Chandler classification which has been updated with, a minor revision. Specifically, as previously reported [3], Chandler type I OC (pre‐septal cellulitis/abscess) presents different treatment modalities and outcomes in the two scenarios of cellulitis and abscess, thus we propose to subdivide it into type Ia (cellulitis) and Ib (abscess), while respecting the rest of the original classification. To validate the modified Chandler classification, we compared its predictive value with that of the original Chandler classification and analyzed the two subclasses (Ia and Ib) with respect to treatment modality, duration of antibiotic therapy and hospitalization, and outcome after primary treatment. Significant differences were found in each aspect (*p* < 0.05, Table 3) not only between the abovementioned classes, but also in a linear‐by‐linear association of all modified Chandler classification, with the primary treatment modality being the most significant variable analyzed (Cramer's *V* = 0.518). Several authors previously underlined that many factors could influence the aggressiveness of the primary treatment, including age, immune deficiency, pretreatment ophthalmologic symptoms and radiologic sinus opacification [14, 17, 18, 19].

To explore factors that may have influenced the upfront treatment approach, a uni/multivariate logistic regression analysis was performed (Table 4): among all data analyzed, the only variables that kept significance at multivariate analysis were the presence of additional complications other than the OC (OR 5.891, 95% CI 1.677–107.507, *p* = 0.015) and the modified Chandler classification (1.743 95% CI 1.349–2.680, *p* < 0.001), with the latter being the most reliable predictor. If the first factor presented a direct trend toward surgery (i.e., the presence of an intra‐cerebral abscess, in addition to the OC, more commonly justified immediate surgery), the interpretation of the second is more complicated. With the exclusion of OC type Ia and V, all other OCs were mainly treated surgically; indeed, the cavernous sinus thrombophlebitis was exceedingly rare (only two patients in the present series), whereas the pre‐septal cellulitis was more frequently treated with an exclusive medical therapy (69%) given the nonsuppurative nature of the infection and its anatomical location anterior to the orbital septum. This fibrotic structure protects the intraconal compartment allowing a less aggressive approach [1, 20]. However, if in the same anatomical region, a purulent collection was identified (type Ib), a more aggressive approach including immediate surgery was adopted (87.5%), regardless of its width and volume: this principle was similar to when a subperiosteal (type III) and intraconal abscess (type IV) were found, with surgery performed respectively in 86.8% and 88.2% of cases. These findings are in line with previous data, in which the surgical approach was preferred in 79.9%–83.3% of type Ib–III–IV OCs [1, 20], and are based on the principle that in cases of abscess formation (typically considered a progression of a cellulitic infection), antibiotic therapy alone is rarely sufficient for a curative effect and surgery may guarantee release pressure in the orbit, reduce the bacterial load and allow for the collection of microbiological samples, which are essential for targeted antibiotic treatment [1]. Of note, this management strategy markedly differs from the pediatric setting, where Chandler I–II and selected III infections (< 5 mm) can often be treated conservatively depending on abscess size and clinical course, whereas in adults a surgical approach is more frequently required due to the lower responsiveness to exclusive medical therapy [8].

Similarly to OC type Ia, orbital cellulitis (type II) was historically known to be treated with an exclusive medical therapy due to its nonsuppurative nature [21, 22]; nevertheless, this infection develops inside a close and delicate compartment with limited space, so that it may cause elevation of the intraconal pressure with potential severe consequence on the visual acuity and orbit integrity. Considering the severity of the infection, in our series 69.2% of cases were treated surgically, with 9 cases of medial orbital decompression, and 16 endoscopic drainages of the endonasal infection. This evidence is a novelty in the literature and should be explained by two main reasons: (1) in cases of visual impairment, the surgical strategy had the goal to decompress the orbital cavity though releasing the orbital pressure and treating the ophthalmologic symptom, rather than directly evacuating the infection; (2) the endoscopic drainage of the pathological sinus had the goal to reduce the bacterial load (independently to the ophthalmologic symptoms), though enabling a more effective action of the antibiotic therapy.

Independently of the OC type, if surgery was indicated, it was generally performed through an endoscopic endonasal approach, which could have been combined, in selected cases, with an external one, based on the abscess location. Specifically, in the case of type Ib or type III OC (located laterally or superiorly), an adjuvant external incision was generally required, whereas in the case of inferior/medial subperiosteal abscess or type IV OC, an exclusive endoscopic approach was preferred. Center‐specific expertise and patients characteristics have likely played a role in the decision‐making process [21, 23].

Independent to the abscess type and location, it should be noted that respectively in 23.4% and 30.1% of cases, a drain inside the pathological sinus and orbital abscess was placed: this surgical management is a novelty in the literature and it is based on the concept to deliver post‐operative rising of the infected cavity and to drain accumulated fluids, thus facilitating the healing process. However, further studies are needed to validate this practice due to the heterogeneity of data.

The resolution of the infection after the primary treatment was investigated in a logistic regression analysis and demonstrated, at univariate analysis, how the modified Chandler classification, INCS and preadmission NSAIDs influenced the resolution of the infection (Table 5), contrary to other factors (i.e., immunocompromised disease, odontogenic infection, and underlying CRS) decided a priori based on available data [3, 18, 24]. When a multivariate model was applied, only the INCS application (OR 3.028, 95% CI 1.070–8.573, *p* = 0.037) kept significance highlighting the protective role of this local medical therapy in the resolution of the infection after primary treatment. Nowadays, there is a lack of evidence regarding the efficacy of such intranasal medications in the OCs management, however, their use seems beneficial in the treatment of ARS by reducing the mucosal inflammation and, thereby, facilitating sinus drainage and reducing the bacterial load [14]. Differently, the use of systemic corticosteroids was not investigated in this manuscript given their not homogeneous use (in terms of indication, molecule, and dose). A recent meta‐analysis found that their application may decrease orbital inflammation with a low risk of exacerbating infection [21]. Nevertheless, this conclusion should be considered carefully due to the lack of sound data in the literature.

The abovementioned protocol of treatment based on the modified Chandler classification guaranteed uniformed long‐term ophthalmologic outcomes, with four cases of permanent diplopia (1.9%) and five cases of visual impairment (2.3%), which are lower percentages compared to previous data concerning the type II–III OCs in adulthood (2.5%–6%) [1, 3, 22]. Nevertheless, it should be noted that among all OCs, the number of surgeries varied significantly (*p* < 0.001, Cramer's *V* 0.567), with the type Ib (87.5%) and IV (88.1%) being the most operated, and among them, the type Ib being the most commonly operated ≥ 2 times (37.5%). This evidence could be justified by the fact that type Ib OC may occur either as a consequence of ethmoidal or frontal sinusitis, the latter known to be more difficult to treat in case of complicated infection [3].

Of note, as indicated by the homogeneity of the cases, all centers involved shared the same strategy of treatment for OCs of ARS throughout the study period, thus ensuring uniformity in terms of management and outcomes. Nevertheless, several limitations should be acknowledged, including the retrospective design, the small number of Ib–II–V cases, which limited the possibility of identifying predictors of successful medical versus surgical management, the absence of standardization regarding corticosteroid use, and the lack of objective quantification of sinonasal disease severity. In addition, minor differences in clinical decision‐making may still have occurred and represent a potential source of bias. The correlation between abscess volume and clinical decision‐making would have represented a meaningful aspect to investigate; however, due to the potential heterogeneity in volume assessment across centers in a retrospective multicentric study, this parameter was not included to minimize the risk of intrinsic bias.

## 5 Conclusion

This study highlights the potential role of the modified Chandler classification as the key predictor for an upfront treatment decision; specifically, among all OCs, suppurative ones (types Ib, III, and IV) were mostly managed surgically, similarly to the type II OC that showed a high surgical rate (69.2%) regardless its nonsuppurative nature. Even if each OCs presented a different number of surgeries and ATB/hospitalization duration, the final ophthalmologic outcomes obtained in the present case‐series were homogenous across different OC typos and were more favorable than the results previously reported in the previous literature, supporting the efficacy and safety of the diagnostic and therapeutic protocol described herein.

## Funding

The authors have nothing to report.

## Conflicts of Interest

The authors declare no conflicts of interest.

## Data Availability

The data that support the findings of this study are available on request from the corresponding author. The data are not publicly available due to privacy or ethical restrictions.
